# Identification of Prognostic Genes in Gliomas Based on Increased Microenvironment Stiffness

**DOI:** 10.3390/cancers14153659

**Published:** 2022-07-27

**Authors:** Chaang-Ray Chen, Rong-Shing Chang, Chi-Shuo Chen

**Affiliations:** Department of Biomedical Engineering and Environmental Sciences, National Tsing Hua University, Hsinchu 300044, Taiwan; crchen@mx.nthu.edu.tw (C.-R.C.); rschang@mx.nthu.edu.tw (R.-S.C.)

**Keywords:** glioma, stiffness, extracellular matrix, prognosis, FN1, ITGA5, OSMR, NGFR, TCGA

## Abstract

**Simple Summary:**

Glioblastoma multiforme (GBM) is the most aggressive primary brain cancer; less than 50% of patients with GBM survive longer than 15 months. A biomarker for early GBM diagnosis can substantially increase the effectiveness of therapy for glioma patients. Increased stiffness of brain tumors has been reported during the progression of glioma. In this study, we explored the influences of altered tissue stiffness on gene signaling and its prognosis for glioma patients. We identified four stiffness-dependent genes highly associated with poor prognosis by applying bio-informatics analysis through RNA-Seq and The Cancer Genome Atlas glioma database. Based on pathophysiological observation, the stiffness of the brain tumor was introduced as the key criteria in our meta-analysis of glioma. In addition to the pathophysiology-inspired approach for biomarker identification, our findings provide insights into the relationship between glioma stiffness and prognosis as well as identifying potential molecular treatment targets.

**Abstract:**

With a median survival time of 15 months, glioblastoma multiforme is one of the most aggressive primary brain cancers. The crucial roles played by the extracellular matrix (ECM) stiffness in glioma progression and treatment resistance have been reported in numerous studies. However, the association between ECM-stiffness-regulated genes and the prognosis of glioma patients remains to be explored. Thus, using bioinformatics analysis, we first identified 180 stiffness-dependent genes from an RNA-Seq dataset, and then evaluated their prognosis in The Cancer Genome Atlas (TCGA) glioma dataset. Our results showed that 11 stiffness-dependent genes common between low- and high-grade gliomas were prognostic. After validation using the Chinese Glioma Genome Atlas (CGGA) database, we further identified four stiffness-dependent prognostic genes: FN1, ITGA5, OSMR, and NGFR. In addition to high-grade glioma, overexpression of the four-gene signature also showed poor prognosis in low-grade glioma patients. Moreover, our analysis confirmed that the expression levels of stiffness-dependent prognostic genes in high-grade glioma were significantly higher than in low-grade glioma, suggesting that these genes were associated with glioma progression. Based on a pathophysiology-inspired approach, our findings illuminate the link between ECM stiffness and the prognosis of glioma patients and suggest a signature of four stiffness-dependent genes as potential therapeutic targets.

## 1. Introduction

Glioblastoma multiforme (GBM) is one of the most aggressive primary brain cancers. Patients with glioma are typically treated with surgical resection, chemotherapy, and radiotherapy [1]. Although progress has been made over the past few decades in the development of these therapeutics for GBM, the median survival time remains less than 15 months [2]. Therefore, the identification of effective biomarkers for early detection and diagnosis is urgently required to improve the prognosis and treatment of glioma patients.

With the advent of RNA sequencing and bioinformatics, abundant public sequencing data facilitate the discovery of biomarker genes, regulatory networks, and pathways associated with GBM. Pilot studies conducted by The Cancer Genome Atlas (TCGA) have revealed four subtypes of GBM [3,4]. Genetic and transcriptomic alterations of the biomarker genes EGFR, NF1, PDGFRA/IDH1, and NEFL define the classical, mesenchymal, proneural, and neural subtypes, respectively. In addition, TCGA provides not only genomic profiles but also clinical information, enabling us to predict the prognosis of cancers by using survival analysis. A recent study revealed 22 universal biomarker genes for diagnosis and prognosis across 29 cancers from TCGA, which were linked to the frequently mutated TP53-, MAPK-, PI3K-, and AKT- related pathways [5]. As for glioma, Xu et al. identified a four-gene (OSMR, SOX21, MED10, and PTPRN) signature related to survival and recurrence time by using weighted gene co-expression network analysis [6]. A meta-analysis incorporating TCGA and three public RNA-Seq datasets identified 104 common genes correlated with overall survival between GBM and low-grade gliomas [7]. Moreover, recent studies have revealed prognostic signature genes specific to DNA damage repair and the tumor immune microenvironment [8,9,10,11].

Increasing clinical evidence has indicated that ECM stiffness plays a crucial role in modulating glioma migration, invasiveness, and progression [12,13,14,15]. The stiffness of gliomas gradually increases from several hundred pascals to tens of thousands of pascals, in accordance with aggressiveness [16]. The increased stiffness of the tumor microenvironment (TME) can promote GBM proliferation by enhancing EGFR signaling [17]. A stiffer matrix also upregulates the stemness of glioma cells by activating BCL9L/Wnt/Beta-catenin signaling, resulting in sustained tumor growth [18]. Moreover, ECM stiffness is also associated with treatment resistance, including that of glioma. Erickson et al. showed that large spheroids of U87 GBM cells were formed in stiff scaffolds exhibiting a higher degree of temozolomide (TMZ) drug resistance [19]. Using patient-derived xenograft GBM cells, Zhu et al. demonstrated that ECM stiffness can directly regulate how GBM cells respond to treatment with TMZ [20]. Collectively, genes associated with ECM stiffness could be potential biomarkers for improving treatment. However, the association between ECM stiffness and the potential prognostic value of biomarker genes in clinical settings has not been fully established.

In this study, we explored the association between ECM stiffness and prognosis via bioinformatics analysis. We built a comprehensive list of stiffness-dependent genes from an RNA-Seq dataset and evaluated their prognosis based on both low- and high-grade gliomas in TCGA datasets. Based on further validation using the Chinese Glioma Genome Atlas (CGGA) database [21], we identified FN1, ITGA5, OSMR, and NGFR as stiffness-dependent prognostic genes. Overexpression of these four genes showed poor prognosis in both low- and high-grade gliomas. We also confirmed that the expression levels of stiffness-dependent prognostic genes in high-grade glioma were significantly higher than in low-grade glioma, suggesting that these genes are associated with glioma progression. Our results highlight the link between ECM stiffness and prognosis and provide insights regarding potential molecular treatment targets for gliomas. 

## 2. Materials and Methods

### 2.1. Identification of Stiffness-Dependent Genes

Raw counts from the GSE158097 RNA-Seq dataset [22] for 3D-printed glioma models were downloaded from the National Center for Biotechnology Information GEO database (https://www.ncbi.nlm.nih.gov/geo/ (accessed on 24 May 2022)) [23]. Two different levels of stiffness of ECM regions, 2 kPa and 21 kPa, were applied to capture increased stiffness during glioma progression [22]. We used R package DESeq2 (v1.24.0) with default settings to identify stiffness-dependent or differentially expressed genes (DEGs) between stiff and soft glioma models [24]. We applied the median of ratios method of normalization before differential expression analysis [25]. An adjusted *p* value of <0.05 and fold change (|FC|) > 1.5 were set as the cutoff criteria for DEGs. 

### 2.2. TCGA Glioma Datasets

The RNA sequencing of Level 3 data for high- (TCGA-GBM) and low-grade (TCGA-LGG) glioma were downloaded and processed using the R/Bioconductor pack-age TCGAbiolink [26]. We used DESeq2 (v1.24.0) with default settings to identify DEGs between glioma and normal brain tissue. We performed a median of ratios method of normalization before differential expression analysis. An adjusted *p* value of <0.05 and fold change (|FC|) of >2 were set as the cutoff criteria for DEGs.

### 2.3. Functional Enrichment Analysis

Functional enrichment analysis in Gene Ontology (GO) and KEGG pathways was performed and visualized using the R package clusterProfiler [27]. The category of GO terms was set to Biological Process. An adjusted *p* value of <0.05 was set as the threshold for identification of significantly enriched GO functional terms or KEGG pathways.

### 2.4. Survival Analysis

Prognostic genes were identified using a Kaplan–Meier survival model with survminer (https://github.com/kassambara/survminer (accessed on 24 May 2022)). For each gene, glioma patients were divided into high- (High) and low-expression (Low) groups, using the median expression value as the cutoff. A *p* value of <0.05 was set as the significance threshold of survival analysis.

### 2.5. CGGA Validation Datasets

We downloaded the mRNAseq_693 and mRNAseq_325 datasets for expression profiles and clinical data of glioma patients from the CGGA (http://www.cgga.org.cn (accessed on 24 May 2022)) database (hereafter referred to as CGGA693 and CGGA325, respectively). No normalization was performed on either CGGA dataset. Prognostic genes were identified using a Kaplan–Meier survival model with survminer. A *p* value of <0.05 was set as the significance threshold of survival analysis.

### 2.6. GSE16011 Validation Datasets

The gene expression profiles and clinical data of glioma patients from GSE16011 [22,28] were downloaded from the GEO database. No normalization was performed on either GSE16011 dataset. Prognostic genes were identified using a Kaplan–Meier survival model with survminer. A *p* value of <0.05 was set as the significance threshold of survival analysis.

### 2.7. Multivariate Cox Regression

The multivariate Cox hazard regression method was used to extract expression-value-based risk scores using the R package “survival.” According to the estimated regression coefficients, a prognostic risk score was calculated for each patient [29,30,31]. Based on the four-gene signature, patients were divided into high-risk and low-risk groups with the median risk score as the threshold. A Kaplan–Meier survival analysis was performed to estimate and compare the survival of patients in independent cohorts with high or low scores.

## 3. Results

### 3.1. Identification of Stiffness-Dependent Genes from 3D-Printed Glioma Models

**Figure 1:**
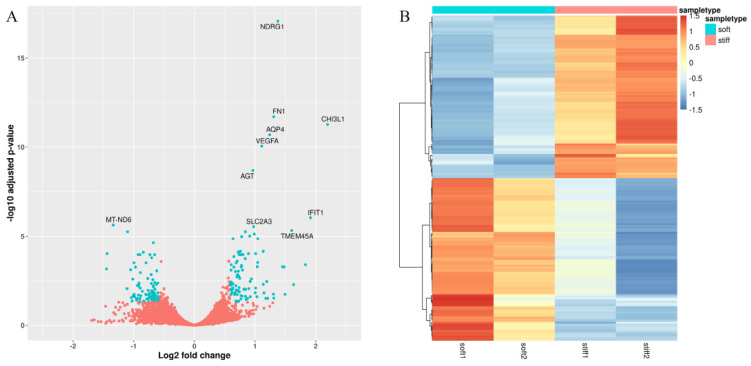
Volcano and heatmap of stiffness−dependent genes. (**A**) Volcano plot for all DEGs. Red dots indicate genes that were not significantly expressed, whereas green dots indicate significantly expressed genes. The 10 most significant DEGs are highlighted with gene names. (**B**) Heatmap of DEGs. Up−regulated genes are denoted by yellow−red colors and down−regulated genes are denoted by navy−blue colors.

To identify genes associated with ECM stiffness, we first compared the expression profiles of stiff (20 kPa) and soft (2 kPa) patient-derived 3D-printed glioma models from the GSE158097 RNA-Seq dataset. Using the soft-ECM model as a reference, the difference in gene expression levels of glioma grown on ECM with two different stiffness conditions was assessed. A significantly differentially expressed gene (DEG) suggests stiffness dependency. After data processing, we identified 190 DEGs as stiffness-dependent genes (Table S1). The 10 most significantly expressed genes were NDRG1, FN1, CHI3L1, AQP4, VEGFA, AGT, IFIT1, MT-ND6, SLC2A3, and TMEM45A (Figure 1A). A total of 95 genes were up-regulated and 95 genes were down-regulated. These results suggest that the genes were up-regulated and down-regulated in the stiff and soft models, respectively (Figure 1B).

### 3.2. Enrichment Analysis of Stiffness-Dependent Genes

**Figure 2:**
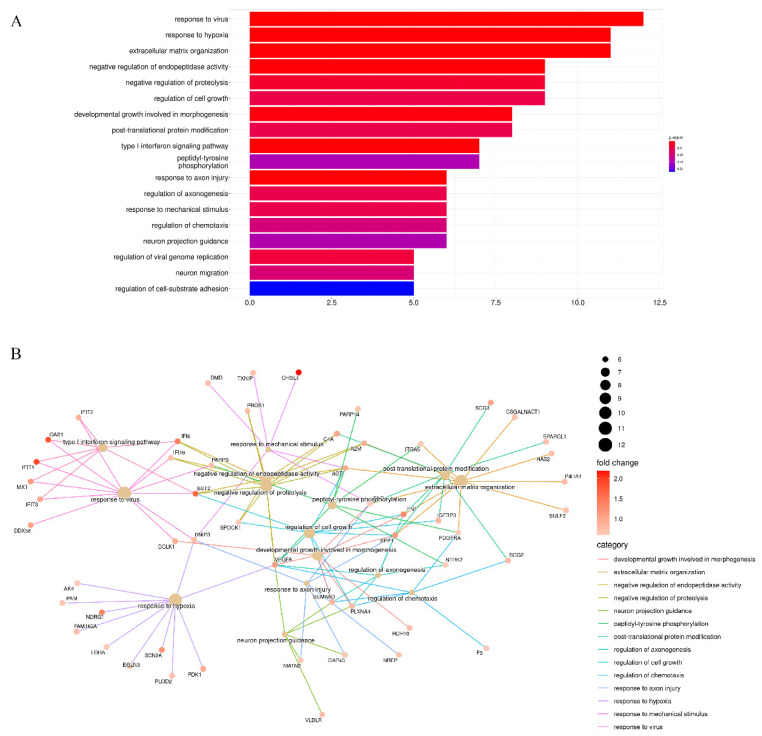
Enriched GO terms for up−regulated stiffness−dependent genes. (**A**) Most enriched GO terms of up−regulated genes. (**B**) Correlation gene networks of up−regulated genes.

To gain further understanding of the biological functions of stiffness-dependent genes, we performed GO enrichment analysis separately for up- and down-regulated genes. The most enriched GO terms of up-regulated stiffness-dependent genes were response to virus, response to hypoxia, ECM organization, negative regulation of endopeptidase activity, negative regulation of proteolysis, and cell growth (Figure 2A). This suggests that these stiffness-dependent genes were activated in response to a stiff TME but repressed in a soft TME. The most enriched GO terms and their corresponding genes were further visualized in inter-connected networks (Figure 2B). Moreover, a KEGG pathway enrichment analysis suggested that HIF-1 signaling was the most crucial pathway for these up-regulated stiffness-dependent genes (Figure S1A).

**Figure 3:**
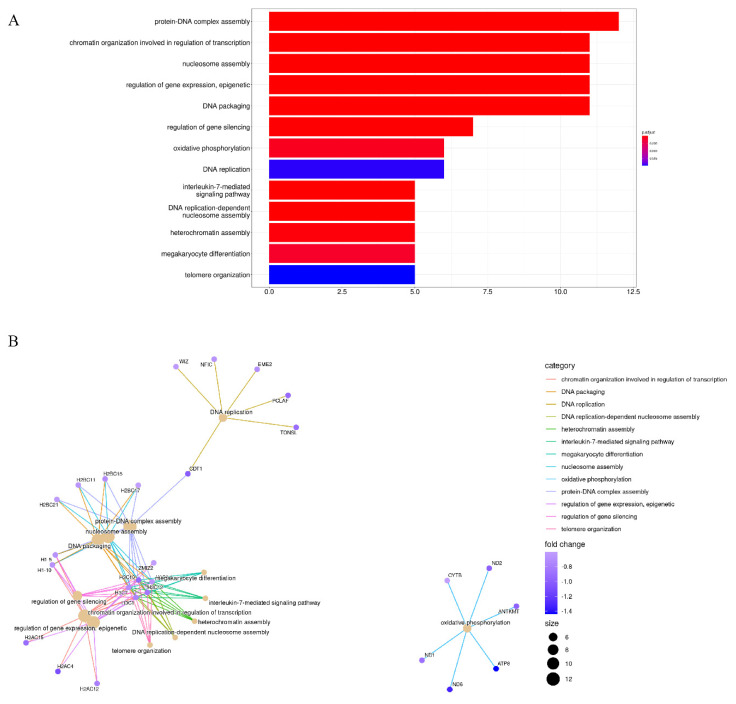
Enriched GO terms for down−regulated stiffness−dependent genes. (**A**) Most enriched GO terms of down−regulated genes. (**B**) Correlation gene networks of down−regulated genes.

Conversely, the results of enrichment analysis revealed distinct GO terms for down-regulated genes, suggesting that these stiffness-dependent genes were activated in response to the soft TME but repressed in the stiff TME. The most enriched GO terms were protein-DNA complex assembly, chromatin organization involved in the regulation of transcription, nucleosome assembly, the regulation of gene expression, epigenetics, and DNA packaging (Figure 3A). The common enriched genes were mostly histone family genes, such as H3C1, H3C2, H3C7, H3C10, and H3C12. Apart from histone family genes, many mitochondrially encoded genes were down-regulated. For example, ND1, ND2, ND6, ATP8, CYTB, and ANTKMT were enriched through oxidative phosphorylation (Figure 3B). Interestingly, KEGG pathway enrichment analysis suggested that systemic lupus erythematosus, alcoholism, and neutrophil extracellular trap formation were the most enriched pathways for these down-regulated stiffness-dependent genes. These pathways are also involved in transcriptional misregulation in cancer, oxidative phosphorylation, and Parkinson’s disease (Figure S1B).

### 3.3. Differential Expression of Stiffness-Dependent Genes in TCGA Glioma Datasets

We downloaded public RNA-Seq expression profiles and clinical data containing low- and high-grade gliomas from TCGA for our analysis. The RNA-Seq data included 156 patients with high-grade glioma (TCGA-GBM), 516 patients with low-grade glioma (TCGA-LGG), and five patients with normal brain tissue. After data-processing, in TCGA-GBM, we identified 13,160 DEGs, including 7223 and 5937 up- and down-regulated genes, respectively. In TCGA-LGG, we identified 6329 DEGs, including 2775 and 3554 up- and down-regulated genes, respectively. By removing genes with inconsistent annotations, 180 stiffness-dependent genes were matched to TCGA datasets. As shown in Table S2, 50 and 90 stiffness-dependent genes were significantly differentially expressed between glioma patients and those with normal brain tissue for TCGA-LGG and TCGA-GBM, respectively.

### 3.4. Survival Analysis of Stiffness-Dependent Genes

**Figure 4:**
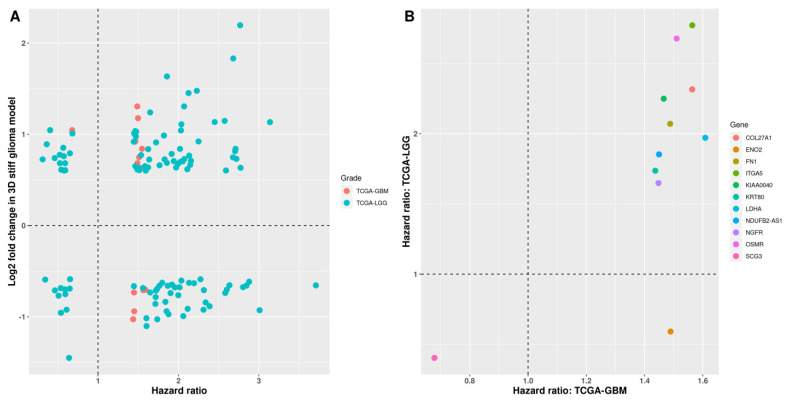
Comparison of hazard ratios between high− and low−grade gliomas. (**A**) Scatterplots of stiffness−dependent gene expression and corresponding hazard ratios for TCGA−GBM and TCGA−LGG. (**B**) Common stiffness−dependent prognostic genes between TCGA−GBM and TCGA−LGG.

A recent study showed that a prognostic gene may not be differentially expressed between various cancers and normal tissue [32]. Therefore, we performed Kaplan–Meier analysis for all stiffness-dependent genes, including those that were not DEGs in TCGA. Our results revealed that 14 and 116 stiffness-dependent genes were prognostic in TCGA-GBM and TCGA-LGG, respectively (Table S3). The expression values of stiffness-dependent genes from GSE158097 were mapped to the corresponding hazard ratio calculated using univariate Cox regression in TCGA. In TCGA-GBM, 13 of 14 stiffness-dependent genes had poor prognosis (hazard ratio > 1) in the high-expression group (Figure 4A). In TCGA-LGG, 90 genes had poor prognosis in the high-expression group, whereas 26 genes had better prognosis (hazard ratio < 1) in the high-expression group. However, no clear correlation was found between the expression values of GSE158097 and the corresponding hazard ratio in either TCGA-GBM or TCGA-LGG.

Collectively, we identified 11 common stiffness-dependent prognostic genes between TCGA-GBM and TCGA-LGG (Figure 4B). Nine high-expression genes showed consistently poor prognosis in both TCGA-GBM and TCGA-LGG, including NGFR, FN1, LDHA, OSMR, ITGA5, KRT80, COL27A1, KIAA0040, and NDUFB2-AS1. The high expression of ENO2 predicted the poor prognosis in TCGA-GBM but better prognosis in TCGA-LGG. Interestingly, the high expression of SCG3 predicted better prognosis in both TCGA-GBM and TCGA-LGG.

### 3.5. Validation Using the CGGA Database

**Figure 5:**
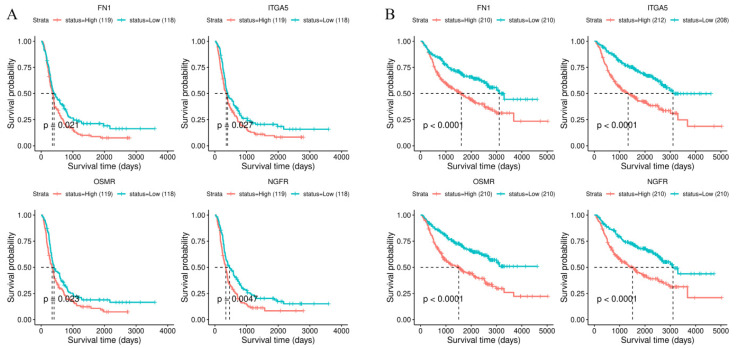
Kaplan–Meier curves of common stiffness−dependent prognostic genes in (**A**) CGGA−GBM and (**B**) CGGA−LGG.

To validate our finding of stiffness-dependent prognostic genes, we examined the independent CGGA database [21]. We constructed CGGA-GBM datasets by including a total of 283 samples from WHO grade IV glioma patients. As for CGGA-LGG, 403 samples from WHO grade II and III glioma patients were included. After Kaplan–Meier analysis, four stiffness-dependent genes were identified from TCGA datasets (FN1, ITGA5, OSMR, and NGFR) as prognostic in CGGA-GBM (Figure 5A). Ten genes (NGFR, FN1, KRT80, HIST1H3F, SCG3, OSMR, KIAA0040, ENO2, ITGA5, and NDUFB2-AS1) were also prognostic in CGGA-LGG (Figure S2). Finally, intersection analysis revealed FN1, ITGA5, OSMR, and NGFR as common genes between CGGA-GBM and CGGA-LGG (Figure 5B). In agreement with the results from TCGA datasets, all genes predicted poor prognosis in the high-expression group.

**Figure 6:**
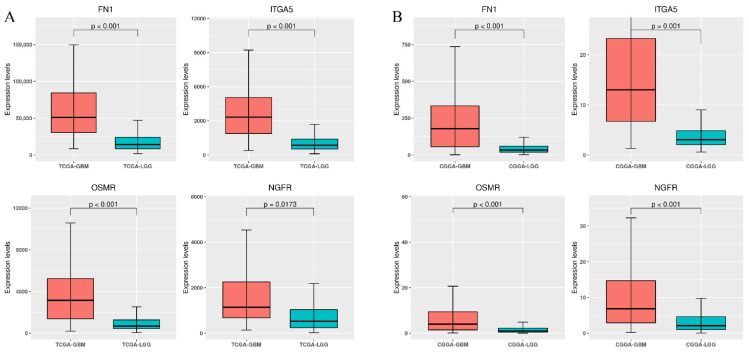
Comparison of gene expression levels between high- and low-grade gliomas for four stiffness-dependent prognostic genes in (**A**) TCGA and (**B**) CGGA. Differences between groups were analyzed using the Student’s *t*-test.

To determine whether these stiffness-dependent prognostic genes were associated with glioma progression, we compared the differential expression between TCGA-GBM and TCGA-LGG. Our results showed that FN1, ITGA5, OSMR, and NGFR were overexpressed in TCGA-GBM (Table S2), suggesting that these genes were not only prognostic but also diagnostic between high-grade gliomas and normal brain tissue. However, only FN1 was overexpressed in TCGA-LGG (Table S2), implying that FN1 could be an early detection biomarker for low-grade glioma patients. Moreover, the expression levels of all four stiffness-dependent prognostic genes in TCGA-GBM were significantly higher than in TCGA-LGG (Figure 6A). As confirmed by the CGGA database, our results showed consistent expression patterns between high- and low-grade gliomas (Figure 6B), demonstrating that these stiffness-dependent prognostic genes were also associated with glioma progression.

### 3.6. Validation of the Four-Gene Signature in Independent Cohorts

**Figure 7:**
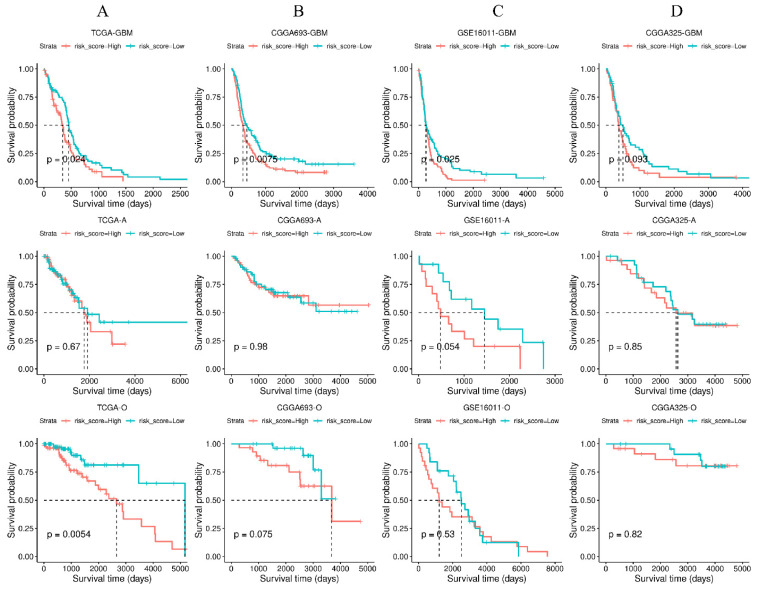
Kaplan–Meier curves of the four stiffness-dependent prognostic gene signatures for four independent cohorts: (**A**) TCGA, (**B**) CGGA_693, (**C**) GSE16011, and (**D**) CGGA_325. GBM: Glioblastoma, O: Oligodendroglioma, A: Astrocytoma.

To assess prognosis based on the four-gene signature, a risk score (r) was calculated using a multivariate Cox regression model for each patient as follows: r = 0.1147 × FN1 + 0.2201 × ITGA5 + 0.2661 × OSMR + 0.2762 × NGFR. The gene symbol indicates the expression value of a gene in TCGA-GBM. Samples were then divided into high- and low-risk groups according to the median of risk scores. Survival analysis with log-rank tests revealed that patients in the low-risk group had a significantly better prognosis than those in the high-risk group (Figure 7A, top).

According to the fifth edition of the WHO Classification of Tumors of the Central Nervous System, published in 2021, adult-type diffuse gliomas are classified into three subtypes: astrocytoma (IDH-mutant), oligodendroglioma (IDH-mutant and 1p/19-codeleted), and glioblastoma (IDH-wildtype) [33]. We further investigated the prognosis of the four-gene signature for astrocytoma and oligodendroglioma from the TCGA-LGG dataset. Our results showed that patients in the low-risk group had a significantly better prognosis than those in the high-risk group for oligodendroglioma (Figure 7A, bottom). However, the survival difference between high- and low-risk groups for astrocytoma was not significant (Figure 7A, middle). As for the CGGA693 dataset, our four-gene signature predicted that patients in the low-risk group had better prognosis than those in the high-risk group for glioblastoma but was not predictive for astrocytoma or oligodendroglioma (Figure 7B). 

We also tested two additional independent datasets: GSE16011 and CGGA325 (see Materials and Methods). In the GSE16011 dataset, our results showed that patients in the low-risk group had better prognosis than those in the high-risk group for glioblastoma but no predictive power was found for astrocytoma or oligodendroglioma (Figure 7C). However, in the CGGA325 dataset, our four-gene signature failed to predict prognosis for all subtypes of diffuse gliomas (Figure 7D). The results of a survival analysis for four independent cohorts are summarized in Table S4.

## 4. Discussion

The mechanical rigidity or stiffness of the ECM is widely known to be crucial in regulating the cellular behavior, invasion, migration, and proliferation of glioma cells [12,13,14,15]. A stiffened ECM has influences on therapeutics, leading to treatment resistance [19,20]. Although several biomarker genes of the ECM have recently been identified, genes associated with ECM stiffness and their prognostic value have not been fully explored. Hence, we performed bioinformatics analyses to link ECM-stiffness-regulated gene expression and prognosis, revealing FN1, ITGA5, OSMR, and NGFR as stiffness-dependent prognostic genes. These genes were not only overexpressed in GBM but also in low-grade gliomas across TCGA and CGGA datasets, highlighting their critical role in glioma progression.

Low-grade gliomas progressed to high-grade gliomas as stiffness gradually increased in accordance with aggressiveness. Of the 180 stiffness-dependent genes identified by our analysis, more than 20% were common DEGs between the TCGA-GBM and TCGA-LGG datasets (Table S2). Whereas extensive efforts have been focused on assessing the effect of ECM stiffness on high-grade gliomas, our results highlight the link between ECM stiffness and low-grade gliomas. Based on the altered expression of the four stiffness-dependent genes, our results provide a valuable resource for further study on the key regulatory genes that mediate cell fate in the progression from low-grade to high-grade glioma. Moreover, our results showed the poor prognosis of the high expression of the four stiffness-dependent genes, suggesting that they are potential therapeutic targets for suppression.

FN1 is a glycoprotein of the ECM that facilitates cell adhesion, growth, migration, and differentiation [34,35,36]. Fibronectins play critical roles in ECM assembly via fibrillogenesis [37,38]. Chen et al. reported that FN1 can be used to diagnose GBM from low-grade astrocytoma, highlighting the crucial role of FN1 in glioma progression and malignancy [39]. In addition to their role as diagnostic biomarkers, the effect of TME-associated genes on therapeutic efficacy has been highlighted in recent studies. Cell adhesion-mediated drug resistance of glioblastoma has been reported [40]. A small molecule inhibitor of TG2 has been found to disrupt the fibronectin matrix assembly, leading to increased sensitivity to chemotherapy [41]. A recent report showed that miR-1 demonstrates tumor suppressive activity in GBM by targeting FN1 [42]. miR-1 expression markedly inhibits tumorigenicity and prolongs animal survival, and FN1 restoration in miR-1-expressing cells in turn restores tumorigenicity, which indicates the critical role of FN1 in glioma progression. Moreover, the high expression of FN1 in the microenvironment can serve as an alternative therapeutic target for drug delivery involving brain tumors [43]. 

The key roles of the integrin family in glioblastoma have been studied intensively, and integrin-α5β3 is known to be a major therapeutic target [44]. ITGA5 and ITGB1 form a receptor for fibronectin and have mainly been explored for their roles in cell-surface mediated signaling. ITGA5 has been reported to affect the invasive nature of many solid tumors by promoting the epithelial mesenchymal transition pathway [45,46]. Because of its correlation with immune infiltration, ITGA5 is also a prognostic gene for gastrointestinal tumors [47]. Recent proteomic analysis has revealed that small interfering RNA knockdown of ITGA5 reduces invadopodia formation in U87MG cells [48]. Moreover, both the epigenetic and transcriptional levels of ITGA5 are effective in predicting TMZ and bevacizumab resistance, revealing the novel roles of ITGA5 in predicting the treatment outcomes of glioma [49]. To eliminate the malignance of GBM with highly expressed integrin-α5β3, various anti-integrin agents, such as RGD-containing peptides, have been developed; RGD peptides can be used as selective carriers to deliver anti-cancer drugs, and have shown strong anti-glioma efficacy [44]. Moreover, because of the high affinity between RGD peptides and integrin, several targeting radiotracers have been developed for phenotypic imaging and radiotherapy [44,50].

The oncostatin M receptor (OSMR) is a member of the type I cytokine receptor family and contributes to the regulation of local immune response and ECM processes in GBM [51,52]. OSMR is also a direct target of up-stream genes and transcription factors. EGFRvIII and OSMR are obligate co-receptors in maintaining oncogenic STAT3 signaling in mouse astrocytes and human brain tumor stem cells [53]. In non-tumor cells, EGFR-OSMR can be activated synergistically by the ligands EGF and OSM [52]. Moreover, the expression of OSMR is linked to resistance to chemotherapy and radiotherapy. The mitochondrial OSRM regulates oxidative phosphorylation and suppression of OSMR in glioblastoma by using a pharmacological inhibitor that improves the response to ionizing radiation and increases survival [54].

The nerve growth factor receptor (NGFR) is a transmembrane protein and cell surface receptor in many human cell types, including some adult brain cells. NGFR-expressing glioma cells in humans enhance migration, induce structural rearrangement of the actin cytoskeleton, and reduce RhoA activity, which are closely related to cell invasion [55]. More importantly, NGFR is required for cancer cell survival and attenuates tumor suppressor p53 through the direct binding of p53 [56]. The depletion of NGFR suppresses human xenograft tumor growth and sensitizes cells to anti-cancer drugs [57,58]. Recently, high NFGR expression has been reported as being associated with immune exclusion of melanoma and the pharmacological inhibitor AG-879, which can restore T cell sensitivity [59]. Although the influence of NFGR inhibition remains to be explored, NGFR may serve as an alternative therapeutic target for glioblastoma. 

Based on pathophysiological observations, the stiffness of the ECM was introduced as the key criteria in our meta-analysis of glioma. We compared the results of our pathophysiology-inspired analysis with those of prognostic genes in other studies (Table S5) [7,8,10,60,61,62,63,64]. On one hand, our data demonstrated an alternative approach to identifying prognostic biomarkers for glioma from a pathophysiological perspective. On the other hand, the prognostic genes identified were also associated with glioma progression based on gene expression levels. Our findings provide insights into the relationship between ECM-stiffness-regulated genes and their prognosis in glioma patients. However, the molecular mechanisms behind the effect of the four-gene signature on mechanical sensing and transduction require further elucidation via in vitro and in vivo experiments.

## 5. Conclusions

In this study, we explored the association between ECM-stiffness-regulated genes and their prognosis in glioma patients. A pathophysiology-inspired approach was adopted to identify prognostic biomarkers related to the stiffness alteration of the TME. Four stiffness-dependent prognostic genes (FN1, ITGA5, OSMR, and NGFR) were identified from GSE158097 and TCGA glioma datasets using bioinformatics analysis. Based on the four stiffness-related signature genes, we built a risk model that predicted a high risk of poor prognosis in glioma patients. Our results implied that ECM stiffness could affect the survival of glioma patients. These stiffness-dependent genes could become therapeutic targets for gliomas. Several pharmacological molecules and adhesive peptides, which can interfere in the mechanical interactions of cells, are suggested to serve as supplementary treatments, although further investigation of how these signature genes are involved in mechanotransduction is required. Our study highlights the importance of the stiffness of the microenvironment in glioma prognosis. In addition to ECM stiffness, we expect more pathophysiology-inspired approaches related to the physical microenvironment will be applied in prognosis analysis in the near future.

## Data Availability

The data presented in this study are available on request from the corresponding author.

## Supplementary Materials

The following supporting information can be downloaded at: https://www.mdpi.com/article/10.3390/cancers14153659/s1. Figure S1: Enriched KEGG terms for (A) up-regulated and (B) down-regulated stiffness-dependent genes. Figure S2: Kaplan–Meier curves of stiffness-dependent prognostic genes in the CGGA for common genes between TCGA-LGG and CGGA-LGG; Table S1: List of 190 differentially expressed genes from GSE158097. Table S2: List of stiffness-dependent and differentially expressed genes from TCGA-GBM and TCGA-LGG; Table S3: List of stiffness-dependent prognostic genes from TCGA-GBM and TCGA-LGG. Table S4: Summary of the results of survival analysis for four independent cohorts. Table S5: Summary of the prognostic genes for GBM or low-grade gliomas in recent studies.
