# Supplementary material for: Identification of Prognostic Genes in Gliomas Based on Increased Microenvironment Stiffness
Source: Cancers (Basel). 2022 Jul 27;14(15):3659. doi: 10.3390/cancers14153659 (PMC9367320; doi:10.3390/cancers14153659)
Supplement: Supplementary file 1 [file cancers-14-03659-s001.zip › cancers-1826555-supplementary.pdf]

Supplementary Materials

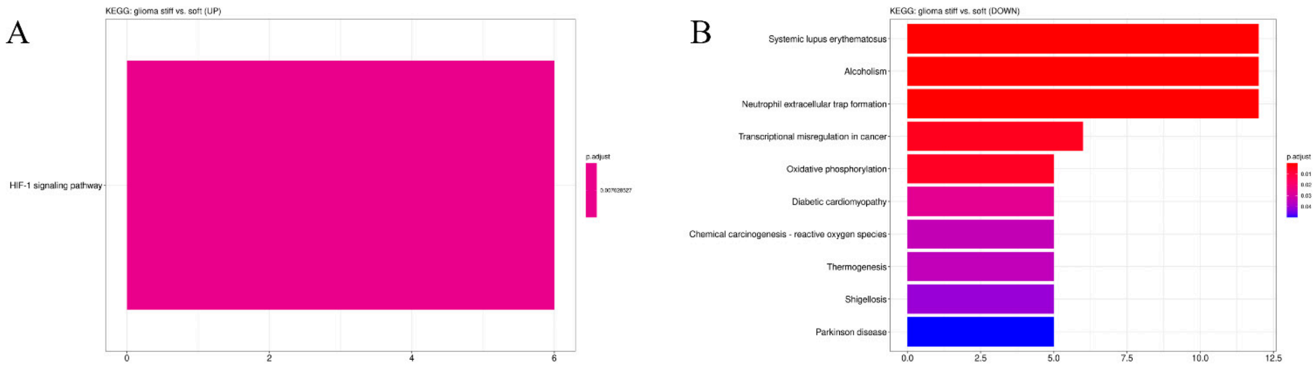

Figure S1. Enriched KEGG terms for (**A**) up-regulated and (**B**) down-regulated stiffness-dependent genes.

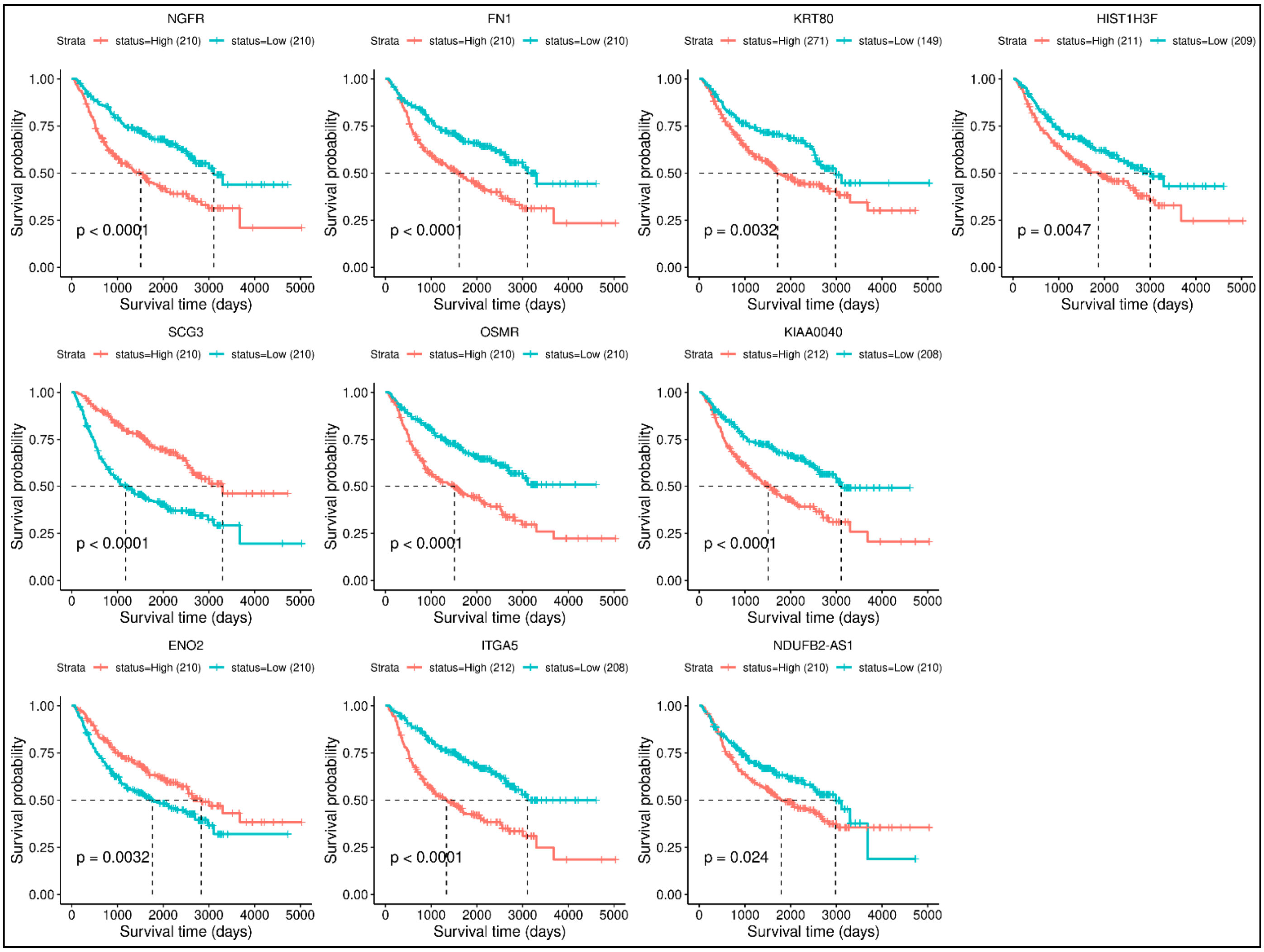

Figure S2. Kaplan–Meier curves of stiffness-dependent prognostic genes in CGGA for the common genes in TCGA-LGG and CGGA-LGG.

Table S1. List of 190 differentially expressed genes from GSE158097.

Ensembl ID	Gene	LFG (log2 Fold Change)	Adjusted P value	Ensembl ID	Gene	LFG (log2 Fold Change)	Adjusted P value
ENSG00000005206	SPPL2B	-0.646772	0.0329397	ENSG00000161638	ITGA5	0.633357	0.00823365
ENSG00000006015	REX1BD	-0.897553	0.0319352	ENSG00000161996	WDR90	-0.884095	0.0220473
ENSG00000007062	PROM1	1.00918	0.000094683	ENSG00000162066	AMDHD2	-0.706101	0.0373431
ENSG00000011451	WIZ	-0.626875	0.0461849	ENSG00000162433	AK4	0.636453	0.00470972
ENSG00000012171	SEMA3B	-0.836142	0.0444711	ENSG00000163328	GPR155	0.627754	0.00966038
ENSG00000034677	RNF19A	0.602468	0.00966038	ENSG00000163565	IFI16	0.677176	0.000970363
ENSG00000041982	TNC	0.603528	0.000801153	ENSG00000163884	KLF15	-0.922529	0.0204121
ENSG00000059804	SLC2A3	0.975258	2.9545E-06	ENSG00000163947	ARHGEF3	0.601495	0.0190061
ENSG00000061938	TNK2	-0.592181	0.0499169	ENSG00000165804	ZNF219	-0.751684	0.0499169
ENSG00000064300	NGFR	-0.733425	0.000107599	ENSG00000166166	TRMT61A	-0.892607	0.00821324
ENSG00000075702	WDR62	-0.674856	0.00720056	ENSG00000166803	PCLAF	-0.922929	0.0063289
ENSG00000078549	ADCYAP1R1	0.776005	0.00044695	ENSG00000167306	MYO5B	0.632335	0.00823365
ENSG00000089127	OAS1	1.83111	0.000402199	ENSG00000167508	MVD	-0.699961	0.0295821
ENSG00000100095	SEZ6L	0.68361	0.043634	ENSG00000167513	CDT1	-1.01573	0.00256172
ENSG00000100311	PDGFB	-0.662556	0.0342422	ENSG00000167601	AXL	-0.972028	0.000544636
ENSG00000100336	APOL4	1.13379	0.00496137	ENSG00000167767	KRT80	-1.02686	0.0441272
ENSG00000101203	COL20A1	-0.621195	0.00087901	ENSG00000170439	METTL7B	0.814964	0.00966038
ENSG00000103254	FAM173A	-0.987981	0.0248465	ENSG00000170961	HAS2	0.72346	0.011403
ENSG00000104112	SCG3	1.04542	1.38206E-05	ENSG00000171885	AQP4	1.24014	2.04602E-11
ENSG00000104419	NDRG1	1.37611	8.41E-18	ENSG00000171951	SCG2	0.799105	0.000106345
ENSG00000104897	SF3A2	-0.746335	0.0186489	ENSG00000172020	GAP43	0.650392	0.000546759
ENSG00000104899	AMH	-1.44831	0.000690057	ENSG00000172366	MCRIP2	-0.934986	0.0316397
ENSG00000105722	ERF	-0.657854	0.0188655	ENSG00000173193	PARP14	0.700247	0.00188932
ENSG00000106009	BRAT1	-0.660019	0.047022	ENSG00000175356	SCUBE2	1.3086	0.0299764
ENSG00000106484	MEST	0.766854	0.0336318	ENSG00000175426	PCSK1	1.17642	0.0309796
ENSG00000106772	PRUNE2	0.587807	0.00496137	ENSG00000175756	AURKAIP1	-0.684136	0.0188655
ENSG00000107201	DDX58	1.03534	0.000522518	ENSG00000175899	A2M	0.911345	9.75502E-06
ENSG00000109107	ALDOC	0.890718	0.000094683	ENSG00000176171	BNIP3	0.760167	7.04252E-05
ENSG00000111674	ENO2	0.683161	0.0060456	ENSG00000180525	PRR26	-0.80197	0.0183821
ENSG00000112715	VEGFA	1.10975	8.96666E-11	ENSG00000181381	DDX60L	0.840754	0.0237577
ENSG00000114019	AMOTL2	-0.769064	0.00235272	ENSG00000181458	TMEM45A	1.60281	4.79025E-06
ENSG00000114023	FAM162A	0.792392	0.000801153	ENSG00000181790	ADGRB1	-0.691105	0.0121155
ENSG00000114268	PFKFB4	0.841321	0.00966038	ENSG00000184221	OLIG1	-0.587721	0.0351261
ENSG00000114480	GBE1	0.665924	0.0149431	ENSG00000184357	HIST1H1B	-0.676455	2.32003E-05
ENSG00000115414	FN1	1.30602	1.97068E-12	ENSG00000184500	PROS1	0.724882	0.000924875
ENSG00000115548	KDM3A	0.607155	0.00974789	ENSG00000184678	HIST2H2BE	-0.627317	0.00496137
ENSG00000117525	F3	0.617649	0.0149672	ENSG00000184897	H1FX	-0.684417	0.0378878
ENSG00000117643	MAN1C1	0.660578	0.0188655	ENSG00000185347	TEDC1	-1.11134	0.00887821

ENSG00000118785	SPP1	1.13439	7.04252E-05	ENSG00000185504	FAAP100	-0.707204	0.0285508
ENSG00000119917	IFIT3	1.00251	0.0152209	ENSG00000185745	IFIT1	1.91285	9.25893E-07
ENSG00000119922	IFIT2	0.875816	0.031024	ENSG00000186193	SAPCD2	-0.710342	0.00447371
ENSG00000121039	RDH10	0.729096	0.0444711	ENSG00000186470	BTN3A2	0.704416	0.0190795
ENSG00000122515	ZMIZ2	-0.606148	0.0407161	ENSG00000187051	RPS19BP1	-0.683297	0.0234504
ENSG00000122584	NXPH1	0.612347	0.0105021	ENSG00000188566	NDOR1	-0.763629	0.0342422
ENSG00000122884	P4HA1	0.778702	1.05376E-05	ENSG00000196562	SULF2	0.725391	8.09843E-05
ENSG00000124635	HIST1H2BJ	-0.655333	0.000107803	ENSG00000196642	RABL6	-0.651044	0.0193519
ENSG00000125520	SLC2A4RG	-0.654379	0.0286886	ENSG00000196739	COL27A1	-0.70779	0.0117649
ENSG00000125534	PPDPF	-0.992653	0.0183821	ENSG00000196756	SNHG17	-0.648988	0.00966038
ENSG00000126461	SCAF1	-0.693781	0.0162332	ENSG00000197136	PCNX3	-0.592462	0.0412095
ENSG00000126464	PRR12	-0.950508	0.031786	ENSG00000197153	HIST1H3J	-0.842424	7.98311E-05
ENSG00000126709	IFI6	1.45198	0.000522518	ENSG00000197774	EME2	-0.701213	0.0248465
ENSG00000128285	MCHR1	1.30221	0.0179864	ENSG00000197785	ATAD3A	-0.740204	0.0226816
ENSG00000129521	EGLN3	1.00929	0.00821324	ENSG00000198695	MT-ND6	-1.33816	2.3507E-06
ENSG00000129911	KLF16	-0.858217	0.0124712	ENSG00000198727	MT-CYB	-0.602791	0.0285508
ENSG00000130055	GDPD2	0.784974	0.0228472	ENSG00000198753	PLXNB3	-0.752783	0.0286351
ENSG00000130303	BST2	1.63424	0.00518247	ENSG00000198763	MT-ND2	-0.99591	0.000311015
ENSG00000130332	LSM7	-0.705587	0.0286442	ENSG00000198888	MT-ND1	-0.896945	0.000107803
ENSG00000130600	H19	0.771921	0.000532065	ENSG00000198947	DMD	0.720227	0.0464597
ENSG00000131095	GFAP	0.606222	0.000402199	ENSG00000205413	SAMD9	1.47685	0.000534556
ENSG00000131584	ACAP3	-0.590022	0.0318091	ENSG00000210196	MT-TP	-0.6785	0.0060059
ENSG00000132361	CLUH	-0.587625	0.0335704	ENSG00000212443	SNORA53	-1.05092	0.000768059
ENSG00000132561	MATN2	0.614537	0.00974789	ENSG00000218891	ZNF579	-1.01007	0.0226816
ENSG00000133048	CHI3L1	2.19492	5.40694E-12	ENSG00000221866	PLXNA4	0.767693	0.0221667
ENSG00000133083	DCLK1	0.896189	0.00890964	ENSG00000221963	APOL6	1.04225	0.000300589
ENSG00000134333	LDHA	0.636074	1.38206E-05	ENSG00000224888	AC138028.2	-0.955685	0.0358656
ENSG00000134815	DHX34	-0.631643	0.0484454	ENSG00000228253	MT-ATP8	-1.43967	0.000094683
ENSG00000134853	PDGFRA	0.60206	0.00426606	ENSG00000231864	AL807752.3	-0.890627	0.00110956
ENSG00000134986	NREP	0.690573	0.0106397	ENSG00000233822	HIST1H2BN	-0.712899	0.00143128
ENSG00000135744	AGT	0.963679	2.08086E-09	ENSG00000235750	KIAA0040	0.921656	0.025231
ENSG00000136295	TTYH3	-0.588856	0.047022	ENSG00000240889	NDUFB2-AS1	-0.93943	0.00584206
ENSG00000136378	ADAMTS7	-0.784208	0.0340533	ENSG00000242802	AP5Z1	-0.738564	0.0162332
ENSG00000136531	SCN2A	1.20833	0.0309856	ENSG00000244731	C4A	1.03002	0.0253067
ENSG00000137872	SEMA6D	0.837441	0.000180364	ENSG00000246859	STARD4-AS1	0.695775	0.0116791
ENSG00000138449	SLC40A1	0.741502	0.0136087	ENSG00000248527	MTATP6P1	-0.955688	0.0381179
ENSG00000138496	PARP9	0.710453	0.00145961	ENSG00000260005	AC027601.1	-0.900337	0.0463808
ENSG00000140450	ARRDC4	0.919084	0.00349432	ENSG00000260892	AC105020.4	-0.826344	0.047022
ENSG00000140983	RHOT2	-0.664416	0.0150999	ENSG00000263740	RN7SL4P	-1.03928	0.0285508

ENSG00000141905	NFIC	-0.677552	0.0333847		ENSG00000265168	AC005726.4	0.852621	0.0356579
ENSG00000142627	EPHA2	-0.6152	0.0236298		ENSG00000265972	TXNIP	0.722808	7.55432E-05
ENSG00000142798	HSPG2	-0.702137	0.0275291		ENSG00000266074	BAHCC1	-0.791246	0.0194432
ENSG00000143847	PPFIA4	1.49427	0.0183821		ENSG00000272114	AL136131.3	1.19191	0.00348904
ENSG00000144481	TRPM8	0.730967	0.00177242		ENSG00000274641	HIST1H2BO	-0.602497	0.00426606
ENSG00000144485	HES6	-0.685502	0.00274083		ENSG00000274997	HIST1H2AH	-0.79715	0.000311015
ENSG00000145623	OSMR	0.747924	0.00230989		ENSG00000275221	HIST1H2AK	-0.916798	0.00509724
ENSG00000145730	PAM	0.687095	0.000311015		ENSG00000275714	HIST1H3A	-0.764232	0.0111561
ENSG00000146674	IGFBP3	0.840062	5.57655E-06		ENSG00000276043	UHRF1	-0.603455	0.0152209
ENSG00000147408	CSGALNACT1	0.735025	0.00775363		ENSG00000277775	HIST1H3F	-0.704576	0.000160661
ENSG00000147852	VLDLR	0.770303	0.000107803		ENSG00000278463	HIST1H2AB	-1.10145	5.57655E-06
ENSG00000148053	NTRK2	0.606449	0.00566567		ENSG00000278828	HIST1H3H	-0.927649	0.000107803
ENSG00000148204	CRB2	-0.660211	0.0275291		ENSG00000281383	FP671120.4	-23.1545	0.000371136
ENSG00000152256	PDK1	0.98658	7.60226E-06		ENSG00000286522		-0.654322	0.00396858
ENSG00000152377	SPOCK1	0.606705	0.0037757		ENSG00000286670		0.939373	0.00251328
ENSG00000152583	SPARCL1	0.739457	0.000107803					
ENSG00000152952	PLOD2	0.706235	0.000246172					
ENSG00000156860	FBRS	-0.664055	0.0162332					
ENSG00000157601	MX1	1.14696	0.0154072					
ENSG00000159200	RCAN1	0.65128	0.000137839					
ENSG00000160949	TONSL	-0.912327	0.00426606					

Table S2. List of 90 and 50 stiffness-dependent DEG from TCGA-GBM (A) and TCGA-LGG (B) respectively.

Table S2 (A.1)

		GSE158097		TCGA-GBM	
Ensembl ID	Gene	log2 Fold Change	Adjusted P value	log2 Fold Change	Adjusted P value
ENSG00000007062	PROM1	1.00918	0.000094683	1.91365	0.00566561
ENSG00000012171	SEMA3B	-0.836142	0.0444711	-2.28487	1.15816E-06
ENSG00000041982	TNC	0.603528	0.000801153	3.75386	1.76841E-15
ENSG00000059804	SLC2A3	0.975258	2.9545E-06	-1.48535	0.00622916
ENSG00000061938	TNK2	-0.592181	0.0499169	-1.33052	0.00176507
ENSG00000064300	NGFR	-0.733425	0.000107599	1.43204	0.0319727
ENSG00000075702	WDR62	-0.674856	7.20E-03	1.60603	0.00101011
ENSG00000089127	OAS1	1.83111	0.000402199	2.63237	0.000139346
ENSG00000100336	APOL4	1.13379	0.00496137	4.47017	1.21462E-11
ENSG00000101203	COL20A1	-0.621195	0.00087901	2.34484	0.0288603
ENSG00000104112	SCG3	1.04542	1.38206E-05	-1.27661	0.0372205
ENSG00000104899	AMH	-1.44831	0.000690057	2.10643	0.00507615
ENSG00000106009	BRAT1	-0.660019	0.047022	1.1096	5.32625E-06
ENSG00000106484	MEST	0.766854	0.0336318	3.61588	2.66752E-11
ENSG00000111674	ENO2	0.683161	0.0060456	-2.32161	4.15842E-09
ENSG00000112715	VEGFA	1.10975	8.96666E-11	2.91819	5.57293E-06
ENSG00000114019	AMOTL2	-0.769064	0.00235272	-1.19892	0.0241933
ENSG00000114268	PFKFB4	0.841321	0.00966038	1.24297	0.00191261
ENSG00000114480	GBE1	0.665924	0.0149431	1.91626	4.86888E-10
ENSG00000115414	FN1	1.30602	1.97068E-12	3.36834	1.02305E-11
ENSG00000118785	SPP1	1.13439	7.04252E-05	2.46026	0.000317876
ENSG00000121039	RDH10	0.729096	0.0444711	2.99024	1.32434E-07
ENSG00000122884	P4HA1	0.778702	1.05376E-05	1.45361	5.42739E-05
ENSG00000124635	HIST1H2BJ	-0.655333	0.000107803	1.80143	0.00168718
ENSG00000125520	SLC2A4RG	-0.654379	0.0286886	1.235	0.000633322
ENSG00000126461	SCAF1	-0.693781	0.0162332	-1.1593	9.66259E-07
ENSG00000126464	PRR12	-0.950508	0.031786	-1.13231	9.71064E-05
ENSG00000126709	IFI6	1.45198	0.000522518	1.94976	0.00595122
ENSG00000129911	KLF16	-0.858217	0.0124712	-1.28824	7.56172E-07
ENSG00000130055	GDPD2	0.784974	0.0228472	1.47547	0.0136167
ENSG00000130303	BST2	1.63424	0.00518247	1.96041	0.00128518
ENSG00000130332	LSM7	-0.705587	0.0286442	1.61058	6.83134E-07
ENSG00000130600	H19	0.771921	0.000532065	7.05874	2.44947E-10
ENSG00000132361	CLUH	-0.587625	0.0335704	-1.0072	3.70612E-05
ENSG00000132561	MATN2	0.614537	0.00974789	1.77941	0.000025139
ENSG00000133048	CHI3L1	2.19492	5.40694E-12	6.01433	5.31618E-12
ENSG00000133083	DCLK1	0.896189	0.00890964	-1.5089	0.000716961
ENSG00000136531	SCN2A	1.20833	0.0309856	-4.23698	6.3894E-22
ENSG00000142798	HSPG2	-0.702137	0.0275291	2.44458	3.38767E-06
ENSG00000143847	PPFIA4	1.49427	0.0183821	-2.85493	1.28042E-08
ENSG00000145623	OSMR	0.747924	0.00230989	2.44804	2.62781E-06
ENSG00000147408	CSGALNACT1	0.735025	0.00775363	-1.23712	0.00107385
ENSG00000152377	SPOCK1	0.606705	0.0037757	-2.9355	7.36039E-06
ENSG00000152952	PLOD2	0.706235	0.000246172	2.00467	1.55029E-06
ENSG00000159200	RCAN1	0.65128	0.000137839	1.40094	0.0170377
ENSG00000160949	TONSL	-0.912327	0.00426606	1.04975	0.0209624
ENSG00000161638	ITGA5	0.633357	0.00823365	2.83003	4.29877E-10
ENSG00000161996	WDR90	-0.884095	0.0220473	1.00722	0.0320755

Table S2 (A.2)

		GSE158097		TCGA-GBM	
Ensembl ID	Gene	log2 Fold Change	Adjusted P value	log2 Fold Change	Adjusted P value
ENSG00000163565	IFI16	0.677176	0.000970363	2.15468	1.30512E-07
ENSG00000166803	PCLAF	-0.922929	0.0063289	5.27491	1.44488E-29
ENSG00000167508	MVD	-0.699961	0.0295821	-1.12392	0.000101935
ENSG00000167513	CDT1	-1.01573	0.00256172	2.36812	4.61576E-08
ENSG00000170961	HAS2	0.72346	0.011403	4.01684	2.1771E-16
ENSG00000175426	PCSK1	1.17642	0.0309796	-1.95954	0.00764624
ENSG00000184357	HIST1H1B	-0.676455	2.32003E-05	3.83134	0.000928691
ENSG00000198753	PLXNB3	-0.752783	0.0286351	-1.50577	0.00182309
ENSG00000205413	SAMD9	1.47685	0.000534556	2.38797	4.38069E-06
ENSG00000235750	KIAA0040	0.921656	0.025231	3.11643	1.56527E-11
ENSG00000240889	NDUFB2-AS1	-0.93943	0.00584206	-1.15011	6.80269E-08
ENSG00000265972	TXNIP	0.722808	7.55432E-05	1.69479	1.30471E-06
ENSG00000274997	HIST1H2AH	-0.79715	0.000311015	3.65415	0.00503172
ENSG00000136378	ADAMTS7	-0.784208	0.0340533	2.67054	1.91692E-05
ENSG00000138449	SLC40A1	0.741502	0.0136087	2.71427	2.80338E-08
ENSG00000138496	PARP9	0.710453	0.00145961	2.21341	2.44805E-10
ENSG00000142627	EPHA2	-0.6152	0.0236298	1.35653	0.0143867
ENSG00000144481	TRPM8	0.730967	0.00177242	2.91319	0.000182376
ENSG00000144485	HES6	-0.685502	0.00274083	1.93889	0.000804928
ENSG00000146674	IGFBP3	0.840062	5.57655E-06	4.23612	2.61601E-09
ENSG00000147852	VLDLR	0.770303	0.000107803	-1.09682	0.0383259
ENSG00000163328	GPR155	0.627754	0.00966038	-1.54151	7.9758E-08
ENSG00000167767	KRT80	-1.02686	0.0441272	1.84944	0.0208665
ENSG00000170439	METTL7B	0.814964	0.00966038	5.62736	4.34366E-16
ENSG00000173193	PARP14	0.700247	0.00188932	1.44145	0.000609722
ENSG00000175356	SCUBE2	1.3086	0.0299764	2.34378	0.000945311
ENSG00000175899	A2M	0.911345	9.75502E-06	1.82195	1.41014E-05
ENSG00000181381	DDX60L	0.840754	0.0237577	1.56182	2.08744E-05
ENSG00000181458	TMEM45A	1.60281	4.79025E-06	3.57887	5.31804E-15
ENSG00000184500	PROS1	0.724882	0.000924875	2.9709	2.54975E-10
ENSG00000186470	BTN3A2	0.704416	0.0190795	2.81508	4.19847E-13
ENSG00000196562	SULF2	0.725391	8.09843E-05	1.05593	0.0258885
ENSG00000197153	HIST1H3J	-0.842424	7.98311E-05	4.00971	0.00244374
ENSG00000221866	PLXNA4	0.767693	0.0221667	-1.31161	0.0137881
ENSG00000221963	APOL6	1.04225	0.000300589	1.35531	0.00488477
ENSG00000224888	AC138028.2	-0.955685	0.0358656	-3.05685	3.41393E-13
ENSG00000263740	RN7SL4P	-1.03928	0.0285508	2.53461	0.00215092
ENSG00000274641	HIST1H2BO	-0.602497	0.00426606	3.42653	0.000180328
ENSG00000276043	UHRF1	-0.603455	0.0152209	4.10898	2.75224E-16
ENSG00000277775	HIST1H3F	-0.704576	0.000160661	4.44958	0.00128044
ENSG00000278828	HIST1H3H	-0.927649	0.000107803	3.21157	3.73464E-07
ENSG00000281383	FP671120.4	-23.1545	0.000371136	1.74985	0.00789888

Table S2 (B)

		GSE158097		TCGA-LGG	
Ensembl ID	Gene	log2 Fold Change	Adjusted P value	log2 Fold Change	Adjusted P value
ENSG00000012171	SEMA3B	-0.836142	0.0444711	-1.72758	0.00127918
ENSG00000041982	TNC	0.603528	0.000801153	2.51244	0.000621462
ENSG00000059804	SLC2A3	0.975258	2.9545E-06	-2.57532	1.21271E-05
ENSG00000078549	ADCYAP1R1	0.776005	0.00044695	1.94753	0.000372184
ENSG00000100336	APOL4	1.13379	0.00496137	2.39794	0.0225778
ENSG00000101203	COL20A1	-0.621195	0.00087901	2.71126	0.00415478
ENSG00000104419	NDRG1	1.37611	8.41E-18	-1.52516	0.00328809
ENSG00000104899	AMH	-1.44831	0.000690057	2.85754	0.000116276
ENSG00000106484	MEST	0.766854	0.0336318	1.68103	0.00322013
ENSG00000111674	ENO2	0.683161	0.0060456	-1.78112	9.72547E-05
ENSG00000115414	FN1	1.30602	1.97068E-12	1.49081	0.0193407
ENSG00000129911	KLF16	-0.858217	0.0124712	-1.51385	5.46723E-06
ENSG00000130332	LSM7	-0.705587	0.0286442	1.00288	0.00414396
ENSG00000131095	GFAP	0.606222	0.000402199	1.66123	0.0126838
ENSG00000132561	MATN2	0.614537	0.00974789	1.6831	0.00438854
ENSG00000134333	LDHA	0.636074	1.38206E-05	-1.11258	0.0301914
ENSG00000134853	PDGFRA	0.60206	0.00426606	2.39476	0.000862867
ENSG00000136531	SCN2A	1.20833	0.0309856	-2.47066	0.000635268
ENSG00000138449	SLC40A1	0.741502	0.0136087	1.731	0.000241023
ENSG00000138496	PARP9	0.710453	0.00145961	1.26851	0.0148706
ENSG00000143847	PPFIA4	1.49427	0.0183821	-2.32	0.000244537
ENSG00000144485	HES6	-0.685502	0.00274083	3.39864	4.34554E-11
ENSG00000146674	IGFBP3	0.840062	5.57655E-06	1.93926	0.0348448
ENSG00000147852	VLDLR	0.770303	0.000107803	-1.38842	0.00509711
ENSG00000152377	SPOCK1	0.606705	0.0037757	-1.28557	0.0306853
ENSG00000163565	IFI16	0.677176	0.000970363	1.42065	0.00166114
ENSG00000163884	KLF15	-0.922529	0.0204121	1.48386	0.00210614
ENSG00000163947	ARHGEF3	0.601495	0.0190061	-1.10147	0.00369954
ENSG00000165804	ZNF219	-0.751684	0.0499169	1.17689	0.00237552
ENSG00000166803	PCLAF	-0.922929	0.0063289	3.23985	0.000102451
ENSG00000167513	CDT1	-1.01573	0.00256172	1.40396	0.0321921
ENSG00000170439	METTL7B	0.814964	0.00966038	3.09728	0.0059871
ENSG00000170961	HAS2	0.72346	0.011403	2.78792	0.000221989
ENSG00000173193	PARP14	0.700247	0.00188932	1.21676	0.0188917
ENSG00000175356	SCUBE2	1.3086	0.0299764	2.22524	0.000933381
ENSG00000175426	PCSK1	1.17642	0.0309796	-2.28704	0.026541
ENSG00000175899	A2M	0.911345	9.75502E-06	1.11809	0.0132437
ENSG00000181381	DDX60L	0.840754	0.0237577	1.09317	0.0369805
ENSG00000181458	TMEM45A	1.60281	4.79025E-06	1.97547	6.30569E-05
ENSG00000184221	OLIG1	-0.587721	0.0351261	1.38461	0.0111456
ENSG00000184500	PROS1	0.724882	0.000924875	1.92135	0.000588733
ENSG00000186193	SAPCD2	-0.710342	0.00447371	1.71068	0.0173937
ENSG00000186470	BTN3A2	0.704416	0.0190795	2.18715	4.39909E-05
ENSG00000196562	SULF2	0.725391	8.09843E-05	2.20894	1.64587E-06
ENSG00000205413	SAMD9	1.47685	0.000534556	1.403	0.0271278
ENSG00000224888	AC138028.2	-0.955685	0.0358656	-1.325	0.00269788
ENSG00000240889	NDUFB2-AS1	-0.93943	0.00584206	-1.38407	5.88451E-07
ENSG00000265972	TXNIP	0.722808	7.55432E-05	2.07805	1.13749E-08
ENSG00000276043	UHRF1	-0.603455	0.0152209	3.76239	5.66531E-14
ENSG00000281383	FP671120.4	-23.1545	0.000371136	-1.92078	0.0189995

Table S3. List of 14 and 116 stiffness-dependent prognostic genes from TCGA-GBM (A) and TCGA-LGG (B).

Table S3 (A)

		GSE158097		Survival analysis of TCGA-GBM (HEG/LEG : high/low expression gene)				Differential expression of TCGA-GBM		
Ensembl ID	Gene	log2 Fold Change	Adjusted P value	P value	HEG Median survival time (days)	LEG Median survival time (days)	Differential survival time (days)	log2 Fold Change	Adjusted P value	DEG in TCGA-LGG
ENSG00000115414	FN1	1.30602	1.97068E-12	0.0323191	357	454	-97	3.36834	1.02305E-11	Y
ENSG00000235750	KIAA0040	0.921656	0.025231	0.0358911	357	448	-91	3.11643	1.56527E-11	Y
ENSG00000161638	ITGA5	0.633357	0.00823365	0.0146435	342	454	-112	2.83003	4.29877E-10	Y
ENSG00000111674	ENO2	0.683161	0.0060456	0.0268753	329	455	-126	-2.32161	4.15842E-09	Y
ENSG00000240889	NDUFB2-AS1	-0.93943	0.00584206	0.0396937	380	460	-80	-1.15011	6.80269E-08	Y
ENSG00000145623	OSMR	0.747924	0.00230989	0.0245268	342	454	-112	2.44804	2.62781E-06	Y
ENSG00000277775	HIST1H3F	-0.704576	0.000160661	0.0157875	384	505	-121	4.44958	0.00128044	Y
ENSG00000114268	PFKFB4	0.841321	0.00966038	0.0173723	357	468	-111	1.24297	0.00191261	Y
ENSG00000175426	PCSK1	1.17642	0.0309796	0.026107	360	454	-94	-1.95954	0.00764624	Y
ENSG00000134333	LDHA	0.636074	1.38206E-05	0.00976214	357	454	-97	0.999223	0.00772548	Y
ENSG00000167767	KRT80	-1.02686	0.0441272	0.0480174	360	454	-94	1.84944	0.0208665	Y
ENSG00000064300	NGFR	-0.733425	0.000107599	0.0421853	360	460	-100	1.43204	0.0319727	Y
ENSG00000104112	SCG3	1.04542	1.38206E-05	0.0331176	455	384	71	-1.27661	0.0372205	Y
ENSG00000196739	COL27A1	-0.70779	0.0117649	0.0134037	360	480	-120	0.578598	0.406699	N

Table S3 (B.1)

		GSE158097		Survival analysis of TCGA-LGG (HEG/LEG : high/low expression gene)				Differential expression of TCGA-LGG		
Ensembl ID	Gene	log2 Fold Change	Adjusted P value	P value	HEG Median survival time (days)	LEG Median survival time (days)	Differential survival time (days)	log2 Fold Change	Adjusted P value	DEG in TCGA-LGG
ENSG00000144481	TRPM8	0.730967	0.00177242	2.29066E-07	1886	2988	-1102	0.472833	0	N
ENSG00000144485	HES6	-0.685502	0.00274083	0.000559427	2875	1915	960	3.39864	4.34554E-11	Y
ENSG00000240889	NDUFB2-AS1	-0.93943	0.00584206	0.000748022	1886	2988	-1102	-1.38407	5.88451E-07	Y
ENSG00000196562	SULF2	0.725391	8.09843E-05	2.8241E-10	3571	1666	1905	2.20894	1.64587E-06	Y
ENSG00000129911	KLF16	-0.858217	0.0124712	0.00294733	1933	2835	-902	-1.51385	5.46723E-06	Y
ENSG00000059804	SLC2A3	0.975258	2.9545E-06	0.0394378	2235	2907	-672	-2.57532	1.21271E-05	Y
ENSG00000186470	BTN3A2	0.704416	0.0190795	8.06566E-05	1666	2988	-1322	2.18715	4.39909E-05	Y
ENSG00000111674	ENO2	0.683161	0.0060456	0.00362915	3200	2286	914	-1.78112	9.72547E-05	Y
ENSG00000126461	SCAF1	-0.693781	0.0162332	0.00257733	1915	3200	-1285	-0.997752	9.74412E-05	Y
ENSG00000166803	PCLAF	-0.922929	0.0063289	7.73025E-06	1666	2988	-1322	3.23985	0.000102451	Y
ENSG00000104899	AMH	-1.44831	0.000690057	0.0164782	2835	2052	783	2.85754	0.000116276	Y
ENSG00000170961	HAS2	0.72346	0.011403	0.00850503	2000	4412	-2412	2.78792	0.000221989	Y
ENSG00000078549	ADCYAP1R1	0.776005	0.00044695	0.000354125	2907	2052	855	1.94753	0.000372184	Y
ENSG00000106009	BRAT1	-0.660019	0.047022	0.00181624	1933	2907	-974	0.961654	0.000555578	Y
ENSG00000184500	PROS1	0.724882	0.000924875	0.00110596	2052	3470	-1418	1.92135	0.000588733	Y
ENSG00000041982	TNC	0.603528	0.000801153	7.23167E-07	1886	4084	-2198	2.51244	0.000621462	Y
ENSG00000134853	PDGFRA	0.60206	0.00426606	0.00237715	2875	1886	989	2.39476	0.000862867	Y
ENSG00000012171	SEMA3B	-0.836142	0.0444711	0.000748315	1933	2875	-942	-1.72758	0.00127918	Y
ENSG00000163565	IFI16	0.677176	0.000970363	0.000259711	1933	4068	-2135	1.42065	0.00166114	Y
ENSG00000163884	KLF15	-0.922529	0.0204121	0.00590621	2907	1915	992	1.48386	0.00210614	Y
ENSG00000165804	ZNF219	-0.751684	0.0499169	0.00387805	2907	1891	1016	1.17689	0.00237552	Y
ENSG00000224888	AC138028.2	-0.955685	0.0358656	0.000641988	2988	1886	1102	-1.325	0.00269788	Y
ENSG00000106484	MEST	0.766854	0.0336318	5.27059E-05	1886	2907	-1021	1.68103	0.00322013	Y
ENSG00000132561	MATN2	0.614537	0.00974789	0.0275691	2052	2660	-608	1.6831	0.00438854	Y
ENSG00000170439	METTL7B	0.814964	0.00966038	1.12301E-07	1886	2988	-1102	3.09728	0.0059871	Y
ENSG00000114480	GBE1	0.665924	0.0149431	4.31246E-05	1886	4068	-2182	0.94131	0.00721341	Y
ENSG00000184221	OLIG1	-0.587721	0.0351261	0.0176381	2988	2235	753	1.38461	0.0111456	Y
ENSG00000131095	GFAP	0.606222	0.000402199	0.0227047	2052	2660	-608	1.66123	0.0126838	Y
ENSG00000175899	A2M	0.911345	9.75502E-06	0.00358467	2000	2907	-907	1.11809	0.0132437	Y
ENSG00000138496	PARP9	0.710453	0.00145961	5.06353E-05	1886	2875	-989	1.26851	0.0148706	Y
ENSG00000186193	SAPCD2	-0.710342	0.00447371	3.11793E-05	4068	1891	2177	1.71068	0.0173937	Y
ENSG00000173193	PARP14	0.700247	0.00188932	0.000183426	1915	2835	-920	1.21676	0.0188917	Y
ENSG00000115414	FN1	1.30602	1.97068E-12	0.000120151	2235	4412	-2177	1.49081	0.0193407	Y
ENSG00000176171	BNIP3	0.760167	7.04252E-05	0.00208764	2907	2000	907	-0.771611	0.0199516	Y
ENSG00000100336	APOL4	1.13379	0.00496137	4.92285E-09	1666	4068	-2402	2.39794	0.0225778	Y
ENSG00000205413	SAMD9	1.47685	0.000534556	4.09227E-05	1933	3571	-1638	1.403	0.0271278	Y
ENSG00000134333	LDHA	0.636074	1.38206E-05	0.000297612	1933	2988	-1055	-1.11258	0.0301914	Y
ENSG00000167513	CDT1	-1.01573	0.00256172	0.0102339	2000	2835	-835	1.40396	0.0321921	Y
ENSG00000146674	IGFBP3	0.840062	5.57655E-06	0.000203205	1933	3200	-1267	1.93926	0.0348448	Y
ENSG00000181381	DDX60L	0.840754	0.0237577	9.7706E-08	1585	3571	-1986	1.09317	0.0369805	Y
ENSG00000034677	RNF19A	0.602468	0.00966038	0.0110573	2052	3200	-1148	0.899714	0.061555	N
ENSG00000125534	PPDPF	-0.992653	0.0183821	7.31492E-05	1915	3200	-1285	-0.694784	0.0711228	N
ENSG00000235750	KIAA0040	0.921656	0.025231	2.72002E-05	2052	2988	-936	1.48346	0.0732315	N
ENSG00000221963	APOL6	1.04225	0.000300589	0.000107229	1886	2988	-1102	0.997879	0.0747191	N
ENSG00000104112	SCG3	1.04542	1.38206E-05	6.58097E-07	3200	1585	1615	1.0928	0.0814148	N
ENSG00000265168	AC005726.4	0.852621	0.0356579	0.00163268	3978	1915	2063	2.40546	0.0853321	N
ENSG00000278828	HIST1H3H	-0.927649	0.000107803	2.56457E-08	1666	4412	-2746	1.44169	0.094073	N
ENSG00000148053	NTRK2	0.606449	0.00566567	0.0036152	2875	1933	942	0.89203	0.102157	N

Table S3 (B.2)

		GSE158097		Survival analysis of TCGA-LGG (HEG/LEG : high/low expression gene)				Differential expression of TCGA-LGG		
Ensembl ID	Gene	log2 Fold Change	Adjusted P value	P value	HEG Median survival time (days)	LEG Median survival time (days)	Differential survival time (days)	log2 Fold Change	Adjusted P value	DEG in TCGA-LGG
ENSG00000167306	MYO5B	0.632335	0.00823365	0.0260502	2282	2835	-553	-1.28125	0.104284	N
ENSG00000136378	ADAMTS7	-0.784208	0.0340533	0.00271796	1886	2907	-1021	1.2701	0.114024	N
ENSG00000121039	RDH10	0.729096	0.0444711	6.95406E-05	1915	2835	-920	1.08209	0.11632	N
ENSG00000130600	H19	0.771921	0.000532065	0.0221972	2433	2660	-227	2.49047	0.119563	N
ENSG00000142798	HSPG2	-0.702137	0.0275291	4.25341E-07	1891	3978	-2087	1.19775	0.154456	N
ENSG00000161638	ITGA5	0.633357	0.00823365	7.69033E-08	1886	4412	-2526	0.956005	0.155919	N
ENSG00000089127	OAS1	1.83111	0.000402199	1.40554E-07	1666	3470	-1804	1.2209	0.158553	N
ENSG00000274641	HIST1H2BO	-0.602497	0.00426606	0.00026707	1915	3571	-1656	1.61011	0.158617	N
ENSG00000197153	HIST1H3J	-0.842424	7.98311E-05	0.000135855	2052	4412	-2360	2.20041	0.163488	N
ENSG00000172020	GAP43	0.650392	0.000546759	0.0343392	2286	2660	-374	-1.01809	0.164105	N
ENSG00000152256	PDK1	0.98658	7.60226E-06	0.00100676	1915	2988	-1073	-0.617222	0.164907	N
ENSG00000152952	PLOD2	0.706235	0.000246172	0.000293392	2000	2875	-875	0.698601	0.173069	N
ENSG00000114023	FAM162A	0.792392	0.000801153	0.017731	3571	2000	1571	-0.438719	0.177154	N
ENSG00000136295	TTYH3	-0.588856	0.047022	1.21149E-05	1585	2988	-1403	-0.630852	0.2001	N
ENSG00000161996	WDR90	-0.884095	0.0220473	2.66813E-06	1666	3200	-1534	0.615843	0.209506	N
ENSG00000133048	CHI3L1	2.19492	5.40694E-12	9.31232E-08	1886	2988	-1102	2.06554	0.215039	N
ENSG00000007062	PROM1	1.00918	0.000094683	0.0404141	2286	2835	-549	0.831525	0.227249	N
ENSG00000140450	ARRDC4	0.919084	0.00349432	0.0426379	2000	2660	-660	0.633648	0.23291	N
ENSG00000244731	C4A	1.03002	0.0253067	0.0321104	2000	3571	-1571	0.982773	0.239078	N
ENSG00000117525	F3	0.617649	0.0149672	5.81083E-05	1886	2907	-1021	-0.951815	0.252124	N
ENSG00000061938	TNK2	-0.592181	0.0499169	9.42238E-09	2988	1666	1322	0.623058	0.259718	N
ENSG00000100095	SEZ6L	0.68361	0.043634	0.000473955	3470	1915	1555	0.853853	0.275962	N
ENSG00000107201	DDX58	1.03534	0.000522518	0.0422123	2282	2660	-378	0.522991	0.27665	N
ENSG00000278463	HIST1H2AB	-1.10145	5.57655E-06	0.0124665	2052	4229	-2177	-1.0887	0.278977	N
ENSG00000152583	SPARCL1	0.739457	0.000107803	2.09295E-05	2988	1666	1322	0.55579	0.2844	N
ENSG00000160949	TONSL	-0.912327	0.00426606	5.00308E-05	1891	2988	-1097	0.52153	0.321772	N
ENSG00000148204	CRB2	-0.660211	0.0275291	0.000586682	1915	3200	-1285	-0.90228	0.321912	N
ENSG00000122584	NXPH1	0.612347	0.0105021	0.000724334	2988	1915	1073	0.730222	0.341857	N
ENSG00000005206	SPPL2B	-0.646772	0.0329397	0.000374141	1891	2907	-1016	0.372885	0.364482	N
ENSG00000145623	OSMR	0.747924	0.00230989	2.80937E-07	1915	3470	-1555	0.695391	0.40026	N
ENSG00000137872	SEMA6D	0.837441	0.000180364	0.009191	2000	2835	-835	-0.420611	0.405807	N
ENSG00000130303	BST2	1.63424	0.00518247	0.000865671	1933	2875	-942	0.631655	0.415275	N
ENSG00000188566	NDOR1	-0.763629	0.0342422	0.000159429	1891	3200	-1309	0.228606	0.421333	N
ENSG00000109107	ALDOC	0.890718	0.000094683	4.49708E-08	3978	1585	2393	0.51197	0.423919	N
ENSG00000162433	AK4	0.636453	0.00470972	0.00870482	2235	2875	-640	-0.551474	0.424563	N
ENSG00000134815	DHX34	-0.631643	0.0484454	1.92604E-05	1666	3200	-1534	-0.241709	0.508105	N
ENSG00000126709	IFI6	1.45198	0.000522518	4.29028E-05	1886	3200	-1314	0.635072	0.519109	N
ENSG00000156860	FBRS	-0.664055	0.0162332	0.0428808	2282	2907	-625	-0.106336	0.572104	N
ENSG00000105722	ERF	-0.657854	0.0188655	4.66531E-08	1585	4084	-2499	-0.231087	0.577345	N
ENSG00000129521	EGLN3	1.00929	0.00821324	0.034894	2907	2052	855	-0.297855	0.583541	N
ENSG00000141905	NFIC	-0.677552	0.0333847	0.000388601	1933	3200	-1267	0.158646	0.602575	N
ENSG00000130055	GDPD2	0.784974	0.0228472	0.000316852	1915	3571	-1656	0.423503	0.617157	N
ENSG00000175756	AURKAIP1	-0.684136	0.0188655	0.0143871	1933	2907	-974	-0.195606	0.625878	N
ENSG00000184357	HIST1H1B	-0.676455	2.32003E-05	0.000301045	2052	2907	-855	0.837001	0.626866	N
ENSG00000117643	MAN1C1	0.660578	0.0188655	0.0020142	1933	2875	-942	-0.386939	0.659172	N
ENSG00000145730	PAM	0.687095	0.000311015	0.00110532	1891	2907	-1016	-0.222061	0.663557	N
ENSG00000064300	NGFR	-0.733425	0.000107599	0.00640924	2286	2988	-702	0.48277	0.674429	N
ENSG00000167601	AXL	-0.972028	0.000544636	0.000825639	2052	4229	-2177	0.186011	0.728931	N
ENSG00000181790	ADGRB1	-0.691105	0.0121155	0.0159193	3470	2052	1418	-0.292653	0.729985	N
ENSG00000142627	EPHA2	-0.6152	0.0236298	7.37971E-08	1762	3470	-1708	0.267333	0.767398	N
ENSG00000184678	HIST2H2BE	-0.627317	0.00496137	5.16607E-05	1891	4229	-2338	-0.179607	0.784573	N
ENSG00000171885	AQP4	1.24014	2.04602E-11	0.00566804	1886	2835	-949	0.245855	0.787135	N
ENSG00000011451	WIZ	-0.626875	0.0461849	0.00150097	1915	3200	-1285	-0.0737834	0.797121	N
ENSG00000075702	WDR62	-0.674856	0.00720056	4.10978E-08	1547	3470	-1923	0.255247	0.798768	N
ENSG00000167508	MVD	-0.699961	0.0295821	0.00462563	4068	2000	2068	-0.109406	0.831354	N
ENSG00000197785	ATAD3A	-0.740204	0.0226816	0.000535535	1891	2907	-1016	0.07621	0.867181	N
ENSG00000114019	AMOTL2	-0.769064	0.00235272	0.00017331	2907	1891	1016	0.0852622	0.89426	N
ENSG00000157601	MX1	1.14696	0.0154072	3.87801E-07	1666	3571	-1905	-0.13126	0.894501	N
ENSG00000124635	HIST1H2BJ	-0.655333	0.000107803	1.11824E-10	1547	4084	-2537	-0.12734	0.895846	N
ENSG00000248527	MTATP6P1	-0.955688	0.0381179	0.000701375	3200	2000	1200	0.0717538	0.913963	N
ENSG00000242802	AP5Z1	-0.738564	0.0162332	3.52991E-07	1585	3200	-1615	-0.0349811	0.919293	N
ENSG00000196739	COL27A1	-0.70779	0.0117649	3.59613E-06	1666	2907	-1241	0.0942264	0.92119	N
ENSG00000125520	SLC2A4RG	-0.654379	0.0286886	1.43213E-07	1891	2907	-1016	0.0467041	0.937534	N
ENSG00000233822	HIST1H2BN	-0.712899	0.00143128	0.00328286	1915	3200	-1285	-0.0466552	0.948569	N
ENSG00000159200	RCAN1	0.65128	0.000137839	0.0132429	2286	2433	-147	-0.0367418	0.955905	N
ENSG00000167767	KRT80	-1.02686	0.0441272	0.0028929	1933	2907	-974	0.0316043	0.986448	N
ENSG00000118785	SPP1	1.13439	7.04252E-05	1.26956E-06	1762	3571	-1809	-0.00864045	0.99401	N
ENSG00000112715	VEGFA	1.10975	8.96666E-11	8.56009E-05	1891	3200	-1309	0.00289938	0.997415	N

Table S4. Summary of the results of survival analysis for four independent cohorts

Y : p value < 0.05 ; N : p value > 0.05	TCGA	CGGA_693	GSE16011	CGGA_325
GBM (Glioblastoma)	Y	Y	Y	N
A (Astrocytoma)	N	N	N	N
O (Oligodendroglioma)	Y	N	N	N

Table S5. Summary of the prognostic genes for GBM or low-grade gliomas in recent studies

Tumor type	*Database source*	*Sample size*	*Prognostic genes*	*Description of study*	*Reference*
Primary Glioblastoma	GEO, TCGA	955	IGFBP2, PTPRN, STEAP2, SLC39A10	Meta-analysis for predicting survival in primary GBM.	Prasad et al.[60]
Glioblastoma/ Low grade glioma	GEO, TCGA	1089	104 genes; CTSZ, EFEMP2, ITGA5, KDELR2, MDK…	Identification of survival relevant genes shared between GBM and LGG.	Hsu et al. [7]
Low grade glioma	TCGA,CGGA	615	WEE1, CRTAC1,SEMA4G	Identification of signature in predicting survival of LGG.	Xiao et al. [61]
Glioblastoma / Low grade glioma	TCGA,CGGA	1311	17 genes; PARVB, RAP1B, PIK3CA, PGF, VEGFA…	Build a risk signature to predict glioma prognosis using ECM-related gene	Liu et al.[62]
Low grade glioma	TCGA	514	25 genes; HRH3, APLNR, FCER1G, SYK, GNG12…	Identification of prognostic hub genes associated with tumor microenvironment in LGG.	Ni et al. [63]
Glioblastoma / Low grade glioma	CGGA	693	15 genes; ABCC3, COL4A1, PDPN, ANXA1, IGFBP2…	To develop a signature associated with the tumor immune microenvironment	Gong et al. [64]
Glioblastoma / Low grade glioma	GEO, TCGA,CGGA	1431	CDK4, HMGB2, WEE1, SMC3, GADD45G	Construct DNA damage repair genes signature for glioma treatment	Wang et al. [11]
Glioblastoma / Low grade glioma	TCGA	657	TAGLN2, PDPN, TIMP1, EMP3	Prognosis analysis of signature related to tumor immune microenvironment	Lin et al. [8]
Glioblastoma / Low grade glioma	GEO, TCGA,CGGA	956	FN1, ITGA5, OSMR, NGFR	To identify ECM-stiffness related gene signature in glioma	This study
